# Pharmacological postconditioning with sevoflurane after cardiopulmonary resuscitation reduces myocardial dysfunction

**DOI:** 10.1186/cc10496

**Published:** 2011-10-19

**Authors:** Patrick Meybohm, Matthias Gruenewald, Martin Albrecht, Christina Müller, Karina Zitta, Nikola Foesel, Moritz Maracke, Sabine Tacke, Jürgen Schrezenmeir, Jens Scholz, Berthold Bein

**Affiliations:** 1 Department of Anaesthesiology and Intensive Care Medicine, Schleswig-Holstein University Hospital, Campus Kiel, Schwanenweg 21, D-24105 Kiel, Germany; 2 Department of Veterinary Clinical Sciences, Clinic for Small Animals-Surgery, Justus-Liebig University Giessen, Frankfurter Straße 108, D-35392 Giessen, Germany; 3 Department of Physiology and Biochemistry of Nutrition, Max Rubner-Institut Kiel, Federal Research Institute of Nutrition and Food, Hermann-Weigmann-Straße 1, D-24103 Kiel, Germany

**Keywords:** cardiopulmonary resuscitation, echocardiography, inhalation anesthetics, neurological deficits

## Abstract

**Introduction:**

In this study, we sought to examine whether pharmacological postconditioning with sevoflurane (SEVO) is neuro- and cardioprotective in a pig model of cardiopulmonary resuscitation.

**Methods:**

Twenty-two pigs were subjected to cardiac arrest. After 8 minutes of ventricular fibrillation and 2 minutes of basic life support, advanced cardiac life support was started. After successful return of spontaneous circulation (*N *= 16), animals were randomized to either (1) propofol (CONTROL) anesthesia or (2) SEVO anesthesia for 4 hours. Neurological function was assessed 24 hours after return of spontaneous circulation. The effects on myocardial and cerebral damage, especially on inflammation, apoptosis and tissue remodeling, were studied using cellular and molecular approaches.

**Results:**

Animals treated with SEVO had lower peak troponin T levels (median [IQR]) (CONTROL vs SEVO = 0.31 pg/mL [0.2 to 0.65] vs 0.14 pg/mL [0.09 to 0.25]; *P *< 0.05) and improved left ventricular systolic and diastolic function compared to the CONTROL group (*P *< 0.05). SEVO was associated with a reduction in myocardial IL-1β protein concentrations (0.16 pg/μg total protein [0.14 to 0.17] vs 0.12 pg/μg total protein [0.11 to 0.14]; *P *< 0.01), a reduction in apoptosis (increased procaspase-3 protein levels (0.94 arbitrary units [0.86 to 1.04] vs 1.18 arbitrary units [1.03 to 1.28]; *P *< 0.05), increased hypoxia-inducible factor (HIF)-1α protein expression (*P *< 0.05) and increased activity of matrix metalloproteinase 9 (*P *< 0.05). SEVO did not, however, affect neurological deficit score or cerebral cellular and molecular pathways.

**Conclusions:**

SEVO reduced myocardial damage and dysfunction after cardiopulmonary resuscitation in the early postresuscitation period. The reduction was associated with a reduced rate of myocardial proinflammatory cytokine expression, apoptosis, increased HIF-1α expression and increased activity of matrix metalloproteinase 9. Early administration of SEVO may not, however, improve neurological recovery.

## Introduction

Approximately 1 million sudden cardiac arrests occur each year in the United States and Europe [1]. Although the initial return of spontaneous circulation (ROSC) after cardiac arrest (CA) is achieved in about 30% to 40% of cases, only 10% to 30% of these patients admitted to the hospital are discharged with good neurological outcomes. The rest of these patients die during their hospital stay or survive but with neurological sequelae [2].

Organ dysfunction following successful cardiopulmonary resuscitation (CPR) has been attributed mainly to the so-called postresuscitation disease, which is typically characterized by circulatory failure, brain damage, systemic inflammatory response syndrome and alterations in coagulopathy [3]. Postresuscitation myocardial dysfunction is a critical issue and has been reported in 45% to 60% of successfully resuscitated patients [4,5]. Neurological impairment after successful CPR is due mostly to ischemic and/or hypoxic lesions and secondary reperfusion injury to the brain [6].

Pharmacological postconditioning may offer an attractive opportunity to reduce damage to the myocardium and brain within the postresuscitation period. Although pharmacological postconditioning with the volatile anesthetic isoflurane has been shown to attenuate myocardial injury after acute myocardial infarction [7] and to reduce neurohistopathological injury after focal cerebral ischemia [8], its potential protective properties have not yet been investigated in the setting of global ischemia and reperfusion. We hypothesized that the volatile anesthetic sevoflurane (SEVO), when administered during reperfusion after successful CPR, reduces myocardial dysfunction and neurological deficits.

## Materials and methods

This project was approved by the Animal Investigation Committee of the University Schleswig-Holstein, Campus Kiel, Kiel, Germany, and animals were managed in accordance with the guidelines of the University Schleswig-Holstein, Campus Kiel, Kiel, Germany and the Utstein-style guidelines [9]. All animals received human care in compliance with the *Guide for the Care and Use of Laboratory Animals *published by the National Institute of Health (NIH Publication 88.23, revised 1996). Fully detailed methods and protocols are available in Additional files 1 and 2.

### Animal preparation

In this experimental study, we used 22 healthy Goettinger miniature pigs, ages 2 to 4 years, of both genders and weighing 40 to 50 kg. The animals were fasted overnight but had free access to water. Anesthesia was initiated by intramuscular injection of azaperone (4 mg/kg) and atropine (0.01 mg/kg), completed by ear vein injection of propofol (CONTROL) (1 to 2 mg/kg) and sufentanil (0.2 μg/kg). After endotracheal intubation, pigs were ventilated with a volume-controlled ventilator (Sulla 808-V; Dräger AG, Lübeck, Germany) under the following conditions: fraction of inspired oxygen (FiO_2_) of 0.4 at 20 breaths/minute, tidal volume of 8 ml/kg to maintain normocapnia and positive end-expiratory pressure of 5 cm H_2_O. Ventilation was monitored using an inspired/expired gas analyzer that measures oxygen and end-tidal carbon dioxide volume (suction rate, 200 ml/minute) (M-PRESTN monitor; Datex-Ohmeda Inc, Helsinki, Finland). Total intravenous anesthesia was maintained by continuous infusion of propofol (4 mg/kg/hour) and sufentanil (0.2 μg/kg/hour). No neuromuscular blocking agent was administered in this study. Ringer's solution (10 ml/kg/hour) was administered continuously. A standard lead II electrocardiogram (ECG) was used to monitor cardiac rhythm. To ensure an appropriate depth of anesthesia, we took indirect measurements such as tail-clamping, monitoring of the corneal reflex, and lacrimation, as well as changes in hemodynamics and heart rate. If our assessment suggested inadequate level of anesthesia, additional sufentanil or propofol was injected.

A saline-filled central venous catheter (7-French) was inserted in the right internal jugular vein for drug administration. A thermistor-tipped catheter (4-French) for arterial thermodilution (PULSION Medical Systems SE, Munich, Germany) was inserted percutaneously into the right femoral artery. The arterial catheter was connected to the PiCCO system (PiCCO *plus *version 6.0 software; PULSION Medical Systems SE), and the resulting signal was processed to determine mean arterial blood pressure, heart rate and blood temperature. In addition, the arterial catheter allowed discontinuous measurement of transpulmonary cardiac output by injecting 10 ml of ice-cold saline into the proximal port of the central venous catheter. The mean of three consecutive measurements randomly assigned to the respiratory cycle was used for determination of cardiac output. Correction for cardiac index was made by calculating body surface using the formula described previously for pigs [10]. Intravascular catheters were attached to pressure transducers (Smiths Medical, Kirchseeon, Germany) that were aligned at the level of the right atrium. All catheters were flushed with isotonic saline containing 5 IU/ml heparin at a rate of 3 ml/hour to prevent obstruction. Core body temperature was monitored continuously via the arterial catheter. Normothermic body temperature was maintained at 37.0°C to 38.0°C in all animals with a heating blanket throughout the study period.

### Experimental setting

**Figure 1:**
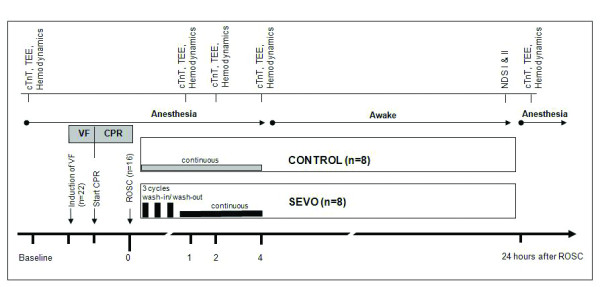
**Experimental time line**. Twenty-two pigs were subjected to cardiac arrest. After 8 minutes of ventricular fibrillation (VF), pigs were resuscitated (cardiopulmonary resuscitation, CPR). Immediately after successful return of spontaneous circulation (ROSC; *N *= 16), the animals were randomized to either (1) total intravenous anesthesia maintained by continuous infusion of propofol (4 mg/kg/hour; CONTROL) or (2) the volatile anesthetic sevoflurane (SEVO) at 1.5 minimum alveolar concentration (3% end-tidal volume) with an initial three interrupted cycles of wash-in (6% end-tidal volume for 5 minutes) and wash-out (0.5% end-tidal volume for 5 minutes) for 4 hours. Neurological function was assessed 24 hours after ROSC by neurological deficit score (NDS) of 1 or 2. Subsequently, animals were again anesthetized using propofol to determine myocardial function by echocardiography, then the animals were killed by an overdose of anesthetic to collect myocardial and cerebral tissue samples. cTnT = cardiac troponin T; TEE = transesophageal echocardiography.

The experimental time line is presented in Figure 1. Following hemodynamic measurements at baseline, ventricular fibrillation (VF) was electrically induced by an alternating current of 5 to 10 V and 1 to 2 mA by a 5-French pacing catheter that was advanced into the right ventricle via the left internal jugular vein, while mechanical ventilation was discontinued. To prevent clot formation, the animals received heparin (100 IU/kg) prior to induction of CA. After an 8-minute nonintervention interval of untreated VF, basic life support CPR was simulated for 2 minutes applying external manual chest compressions at a rate of 100 per minute with a 50% duty cycle, a compression depth of 25% of the anterior-posterior diameter of the chest wall, and a compression-to-ventilation ratio of 30:2. Subsequently, advanced cardiac life support was started with one 100 J biphasic defibrillation attempt (M-Series Defibrillators, Zoll Medical Corp, Chelmsford, USA) according to one-shock protocol. Further, ventilations were performed with 100% oxygen at 20 breaths/minute during CPR. All pigs received 45 μg/kg epinephrine and 0.4 U/kg vasopressin alternating. ROSC was defined as maintenance of an unassisted pulse and a systolic aortic blood pressure of ≥ 60 mm Hg lasting for 10 consecutive minutes according to the Utstein-style guidelines [9]. Since neurological recovery is very unlikely after 30 minutes of normothermic CA, CPR was terminated, when resuscitation remained unsuccessful.

Immediately after ROSC, animals were randomized either to (1) total intravenous anesthesia that was maintained by continuous infusion of propofol (4 mg/kg/h) or (2) the volatile anesthetic SEVO. As we recently found beneficial cardioprotective effects by interrupted SEVO compared with continuous SEVO administration in coronary artery surgery [11], postconditioning was initiated with 3 interrupted cycles of wash-in (6% end-tidal volume for 5 minutes) and wash-out (0.5% end-tidal volume for 5 minutes) followed by a continuous administration of 1.5 minimum alveolar concentration SEVO (3% end-tidal volume) for 4 hours. In both groups sufentanil was administered at a rate of 0.2 μg/kg/h. FiO_2 _was reduced to 0.4 fifteen minutes after ROSC to avoid hyperoxia [12]. Since animals were randomized either to propofol or SEVO not until after ROSC, we used propofol before VF in all animals.

During the initial postresuscitation period, animals received crystalloid infusions to keep mean arterial blood pressure above 50 mm Hg, central venous pressure above 5 mm Hg, and cardiac index at baseline values ± 10%. If this first step failed, additional epinephrine was administered to keep mean arterial blood pressure above 50 mm Hg. We further aimed at serum glucose levels less than 150 mg/dL by intermittent insulin bolus administration. Four hours after ROSC, animals received intramuscular injection of 2 to 3 mg/kg tramadol for pain relief and were weaned from the ventilator. Following extubation, each animal was observed for two hours to ensure adequate spontaneous breathing before being returned to their cages. Twenty-four hours after ROSC, animals were again anesthetized as described above using propofol. After hemodynamic and echocardiographic data were obtained animals were killed by an overdose of sufentanil, propofol and potassium chloride, and tissue samples of the myocardium and cerebral cortex were collected and immediately snap-frozen in liquid nitrogen (stored at -80°C). Autopsy was routinely performed for documentation of potential injuries to the thoracic and abdominal cavity during CPR.

### Measurements

The cumulative defibrillation energy, vasopressor dose, time to ROSC and coronary perfusion pressure were recorded during CPR to ensure comparable ischemia time intervals between groups. Hemodynamic variables, echocardiographic data, cumulative fluid load and cumulative epinephrine dose were recorded at baseline and repetitively up to 24 hours after successful ROSC.

### Echocardiography

Two-dimensional and pulsed wave Doppler transesophageal echocardiography was performed by a single experienced examiner using a Vivid I Cardiovascular Ultrasound System (GE Healtcare, Munich, Germany) with an omniplane probe as described before [13]. The left ventricle end-systolic and end-diastolic volumes were estimated using a four-chamber view by tracing the endocardial border, including the papillary muscles and the method of disks according to the modified Simpson's rule algorithm, then the left ventricular ejection fraction was obtained. For further details, please refer to Additional file 1, Supplemental digital content: methods S1.

### Determination of serum markers and blood gases

Arterial oxygen and carbon dioxide partial pressures and blood glucose levels were measured by using an automatic blood gas analyzer (GEM 4000; Instrumentation Laboratory GmbH, Munich, Germany). For determination of serum markers, arterial blood samples were collected at baseline and 1, 2, 4 and 24 hours after ROSC. Serum was obtained (centrifugation at 3,000 × *g *for 5 minutes) and stored at -20°C until determination of cardiac troponin T by an independent laboratory (Institute of Clinical Chemistry, University Hospital Schleswig-Holstein, Campus Kiel, Kiel, Germany).

### Ventricular arrhythmia

All animals underwent 24-hour ECG recording. Commercially available software was used to detect ventricular arrhythmias (CardioDay Holter ECG; Getemed, Teltow, Germany). All recordings were reviewed and edited by a well-trained technician blinded to the treatment group. The total number of ventricular premature beats, bigeminy, ventricular tachycardia (defined as at least four consecutive complexes lasting at least 120 milliseconds) and VF were counted over an interval of 30 minutes immediately after ROSC and 24 hours later in accordance with the Lambeth Convention [14].

### Neurological evaluation

Overall neurological performance was evaluated at 24 hours after ROSC using two different neurological deficit scores (NDSs 1 and 2) that have been reported previously. The tests consist of different items representing the level of consciousness, respiration, motor and sensory function, posture and feeding behavior. The scores assign different values, depending on the severity of deficits in neurological function, so that a score of 0 is normal and scores of 100 (NDS 1) [15] and 400 (NDS 2) [16] indicate brain death, respectively. Please refer to Additional file 2 which contain two tables showing calculations of NDS 1 (Additional file 2: Table S1) and NDS 2 (Additional file 2: Table S2), respectively.

### Western blot analysis

Caspases are proteases involved in the apoptotic and inflammatory cascade, and caspase-3 in particular is a central mediator of the apoptotic cascade [17]. To quantify apoptosis, we determined uncleaved procaspase-3 by Western blot analysis. In addition, protein expression of hypoxia- and/or ischemia-associated hypoxia-inducible factor (HIF)-1α was evaluated by Western blot analysis. For further details, please refer to Supplemental digital content: methods S1.

### Semiquantitative RT-PCR

Transcript levels of IL-1β, caspase-3, Fas ligand, HIF-1α, matrix metalloproteinase (MMP)-9 and MMP-2 were investigated in myocardial and cerebral cortical tissue and compared between the CONTROL and SEVO groups. For further details, please refer to Supplemental digital content: methods S1.

### ELISA

Protein concentrations of IL-1β were determined by using a swine-specific ELISA system (BioSource International, Inc, Camarillo, CA, USA) in homogenates of myocardial and cerebral tissues according to the manufacturer's protocol and relativized to the amount of total protein in the respective sample.

### Gelatin zymography activity of MMP-9 and MMP-2

Zymography was performed as described previously [13] to detect MMP-9 and MMP-2 activity in myocardial and cerebral tissues. For further details, please refer to Supplemental digital content: methods S1.

### Statistics

Sample size was calculated based on methods described in a previous study [18] to detect a difference with respect to left ventricular ejection fraction of 25% as the primary end point. We calculated a sample size of seven animals in each group to reach an α of 0.05 and power of 80%. To account for animals that did not achieve ROSC or died during the postresuscitation period, we used 11 animals per group. Statistics were performed using commercially available statistical software (GraphPad Prism version 5.02 for Windows; GraphPad Software, San Diego, CA, USA). Data were analyzed by Mann-Whitney *U *test and two-way repeated-measures analysis of variance. In cases where significant differences were observed, the data were adjusted for multiple comparisons (Bonferroni correction). Variables are expressed as means × SD or box-and-whisker plots unless otherwise specified. The figures shows box whiskers with medians [25th to 75th percentile] and the minimum and maximum values (ends of the whiskers). Statistical significance was reached at a two-sided *P *value ≤ 0.05.

## Results

### Survival of animals

Sixteen of twenty-two animals were successfully resuscitated. Detailed resuscitation data are presented in Table 1. Among the animals successfully resuscitated, seven of eight survived for 24 hours in the CONTROL group compared to all eight animals in the SEVO group. One animal in the CONTROL group died as a result of hemodynamic instability during the postresuscitation period.

**Table 1:** Cardiopulmonary resuscitation data

Measurement parameters	CONTROL (*N *= 8)	SEVO (*N *= 8)	*P*-values
24-hour survival rate (*n*)	7	8	1.0^a^
Time to ROSC (minutes)	6.8 ± 1.7	6.6 ± 3.2	0.596
Cumulative epinephrine dose (μg/kg)	30 ± 9	24 ± 11	0.309
Cumulative vasopressin dose (IU/kg)	0.6 ± 0.2	0.5 ± 0.2	0.225
Cumulative defibrillation energy (J)	527 ± 230	528 ± 457	0.595
CorPP (mmHg)	38 ± 9	36 ± 6	0.916

CONTROL = propofol; CorPP = coronary perfusion pressure 10 minutes after induction of ventricular fibrillation; ROSC = return of spontaneous circulation; SEVO = sevoflurane. Data are means ± SD. ^a^Fisher's exact test.

### Myocardial dysfunction and damage

**Figure 2:**
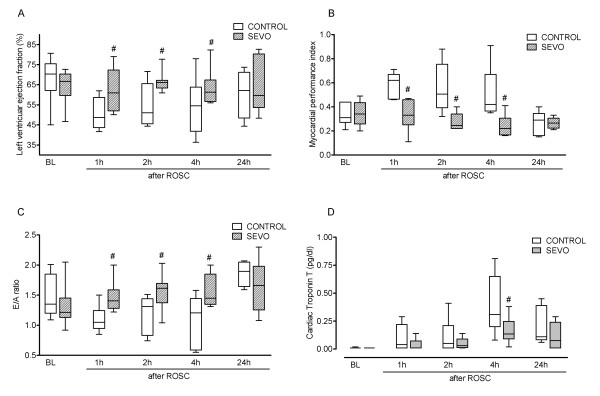
**Myocardial dysfunction and damage**. At baseline (BL) and 1, 2, 4 and 24 hours after return of spontaneous circulation (ROSC), left ventricular ejection fraction **(A)**, myocardial performance index **(B) **and E/A ratio (the ratio between early (diastolic, E) and late (atrial, A) ventricular filling velocity) **(C) **were evaluated by transesophageal echocardiography, and cardiac troponin T serum values **(D) **were quantified in the propofol (CONTROL) and sevoflurane (SEVO) groups. ^#^*P *< 0.05 vs CONTROL.

At baseline, there were no differences with respect to hemodynamic and echocardiographic data, glucose levels and variables of gas exchange. Analysis of echocardiographic data revealed significant (*P *< 0.05) improved left ventricular ejection fraction as the primary end point, myocardial performance index and E/A ratio (the ratio between early (diastolic, E) and late (atrial, A) ventricular filling velocity) in the SEVO group compared with the CONTROL group in the initial period after ROSC, whereas groups did not differ significantly 24 hours after ROSC (Figures 2A through 2C). Animals treated with SEVO had lower peak serum levels of cardiac troponin T 4 hours after ROSC compared with the CONTROL group (*P *< 0.05) (Figure 2D). Further systemic hemodynamic variables are presented in Table 2. Cumulative fluid load and cumulative epinephrine dose within 4 hours following ROSC did not significantly differ between the CONTROL group (fluid = 1,301 ± 412 mL and epinephrine = 30 ± 9 μg/kg) and the SEVO group (fluid = 1,279 ± 374 mL and epinephrine = 24 ± 11 μg/kg) (data not shown). The incidence of ventricular premature beats tended to be decreased in the SEVO group compared to the CONTROL group (Table 3).

**Table 2:** Hemodynamic data

Clinical parameters	HR (beats/minute)	MAP (mmHg)	CVP (mmHg)	CI (L/minute/m^2^)
Baseline				
CONTROL	57 ± 21	88 ± 33	5 ± 2	3.8 ± 0.5
SEVO	65 ± 11	71 ± 14	4 ± 2	3.8 ± 0.7
1-hour ROSC				
CONTROL	116 ± 26	82 ± 8	8 ± 3	4.4 ± 1.3
SEVO	108 ± 11	74 ± 19	7 ± 2	5.1 ± 0.9
2-hour ROSC				
CONTROL	95 ± 19	76 ± 17	6 ± 1	4.4 ± 1.0
SEVO	109 ± 12	81 ± 13	6 ± 1	5.7 ± 1.7
4-hour ROSC				
CONTROL	97 ± 21	76 ± 16	8 ± 2	5.0 ± 1.7
SEVO	101 ± 19	74 ± 13	7 ± 2	5.5 ± 1.6
24-hour ROSC				
CONTROL	75 ± 15	65 ± 9	8 ± 3	4.0 ± 0.8
SEVO	69 ± 15	69 ± 15	7 ± 2	3.4 ± 0.6

Hemodynamic data were determined at baseline and 1, 2, 4 and 24 hours after return of spontaneous circulation (ROSC) in the propofol (CONTROL) and sevoflurane (SEVO) groups. Data are means ± SD. Heart rate (HR), mean arterial pressure (MAP), central venous pressure (CVP) and cardiac index (CI) did not differ significantly between groups after we applied the Bonferroni correction for repeated time measurements using two-way analysis of variance. Cardiac index was calculated as the ratio of cardiac output to body surface area (body surface area = 0.0734 × (body weight in kilograms)^0.656^) [10].

**Table 3:** Ventricular arrhythmias

Clinical parameters	CONTROL	SEVO	*P*-values
**ROSC 30 minutes (*n*)**			
Ventricular premature beats	25 [19 to 41]	20 [13 to 42]	0.601
Bigeminy	1 [1 to 3]	2 [1 to 4]	0.651
VT/VF	-	-	-
**ROSC 24 hours (*n*)**			
Ventricular premature beats	8 [6 to 11]	5 [3 to 10]	0.346
Bigeminy	2 [2 to 5]	2 [1 to 4]	0.584
VT/VF	-	-	-

The total number of ventricular premature beats, bigeminy, ventricular tachycardia (VT)/ventricular fibrillation (VF) ratio was counted over an interval of 30 minutes immediately after return of spontaneous circulation (ROSC) and over a 30-minute interval 24 hours later in the propofol (CONTROL) and sevoflurane (SEVO) groups. Data are medians [25th to 75th percentiles]. Neither ventricular tachycardia nor ventricular fibrillation was observed in any group.

### Cellular mechanisms associated with myocardial dysfunction and damage

**Figure 3:**
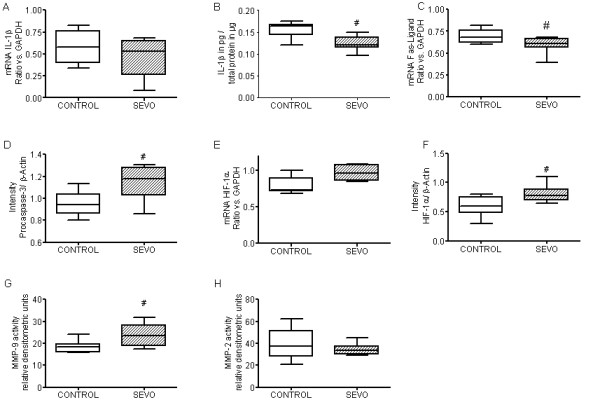
**Cellular mechanisms associated with myocardial dysfunction and damage**. Semiquantitative RT-PCR was used to analyze mRNA expression of IL-1β **(A)**, Fas ligand **(C) **and hypoxia-inducible factor (HIF)-1α **(E) **in myocardial tissue of the propofol (CONTROL) and sevoflurane (SEVO) groups. GAPDH = glyceraldehyde 3-phosphate dehydrogenase. IL-1β protein concentrations were also determined by ELISA **(B)**, and Western blot analysis was used to evaluate protein expression of procaspase 3 **(D) **and HIF-1α **(F)**. Detection of matrix metalloproteinase (MMP)-9 **(G) **and MMP-2 activity **(H) **was performed by gelatin zymography. For further details, please refer to Additional file 1 Supplemental digital content: methods S1. ^#^*P *< 0.05 vs CONTROL.

Compared to the CONTROL group, SEVO reduced both expression of IL-1β mRNA levels (CONTROL vs SEVO: 0.58 arbitrary units (a.u.) [0.4 to 0.76] vs 0.53 a.u. [0.26 to 0.65]; not significant) (Figure 3A) and IL-1β protein concentrations (CONTROL vs SEVO; 0.16 pg/μg total protein [0.14 to 0.17] vs 0.12 pg/μg total protein [0.11 to 0.14]; *P *< 0.01) (Figure 3B). Although mRNA expression of caspase-3 did not differ significantly between the groups (CONTROL 0.87 a.u. [0.82 to 1.11] vs SEVO 1.0 a.u. [0.90 to 1.11]; data not shown), mRNA expression of Fas ligand was significantly decreased in the SEVO group (CONTROL vs SEVO: 0.68 a.u. [0.62 to 0.76] vs 0.61 a.u. [0.56 to 0.67]; *P *< 0.05) (Figure 3C) and uncleaved inactive procaspase-3 was significantly increased in the SEVO group (CONTROL vs SEVO: 0.94 a.u. [0.86 to 1.04] vs 1.18 a.u. [1.03 to 1.28]; *P *< 0.05) (Figure 3D). In addition, both mRNA and protein expression of HIF-1α was increased in the SEVO group (CONTROL vs SEVO mRNA: 0.73 a.u. [0.71 to 0.89] vs 0.96 a.u. [0.85 to 1.07]; not significant; CONTROL vs SEVO protein: 0.60 a.u. [0.48 to 0.75] vs 0.78 a.u. [0.69 to 0.89]; *P *< 0.05) (Figures 3E and 3F). Although mRNA expression of MMP-9 and MMP-2 did not differ between groups (CONTROL vs SEVO MMP-9: 0.07 a.u. [0.04 to 0.29] vs 0.05 a.u. [0.05 to 0.21]; CONTROL vs SEVO MMP-2: 1.31 a.u. [1.25 to 1.67] vs 1.56 a.u. [1.45 to 1.62]; data not shown), SEVO significantly increased MMP-9 activity (CONTROL vs SEVO; 18.5 a.u. [15.9 to 19.7] vs 23.5 a.u. [18.7 to 28.3]; *P *< 0.05) (Figure 3G) and did not affect MMP-2 activity (CONTROL vs SEVO: 37.5 a.u. [27.7 to 51.4] vs 33.6 a.u. [29.7 to 37.3]; not significant) (Figure 3H).

### Neurological dysfunction

**Figure 4:**
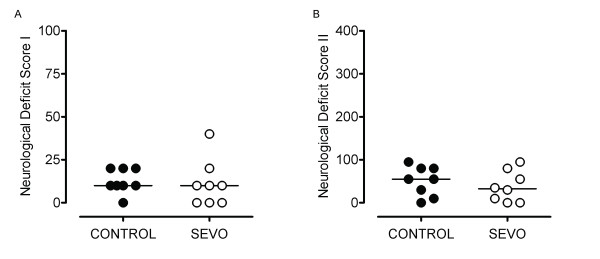
**Neurological deficit scores**. Twenty-four hours after ROSC most of the animals (NDS 1 **(A)**: seven of eight CONTROL animals vs five of eight SEVO animals; NDS 2 **(B)**: seven of eight CONTROL animals vs six of eight SEVO animals) showed neurological deficits, including unsteady gait, irritable consciousness and abnormal respiration. Nevertheless, we did not find any differences between the propofol anesthesia (CONTROL) and sevoflurane anesthesia (SEVO) groups. For further details, please refer to Additional file 2: Supplemental digital content: methods S2.

Twenty-four hours after ROSC, most of the animals (NDS 1: seven of eight in the CONTROL group vs five of eight in the SEVO group; NDS 2: seven of eight in the CONTROL group vs six of eight in the SEVO group) showed neurological deficits, including unsteady gait, irritable consciousness and abnormal respiration. Nevertheless, we did not find any differences between the CONTROL and SEVO groups (Figures 4A and 4B).

### Cellular mechanisms associated with neurological dysfunction

In cerebral cortical tissue, we did not find any difference between the CONTROL and SEVO groups with regard to (1) expression of IL-1β mRNA levels (CONTROL vs SEVO: 0.73 a.u. [0.7 to 0.87] vs 0.79 a.u. [0.73 to 0.84]), (2) IL-1β protein concentrations (0.08 pg/μg total protein [0.08 to 0.1] vs 0.1 pg/μg total protein [0.09 to 0.1]), (3) expression of caspase-3 mRNA (0.94 a.u. [0.82 to 1.04] vs 0.89 a.u. [0.86 to 1.0]), (4) Fas ligand mRNA (0.47 a.u. [0.36 to 0.6] vs 0.41 a.u. [0.34 to 0.51]), (5) uncleaved inactive procaspase-3 (1.27 a.u. [0.29 to 2.56] vs 0.68 a.u. [0.38 to 2.41]) and (6) expression of HIF-1α (mRNA: 1.11 a.u. [1.07 to 1.16] vs 1.19 a.u. [1.11 to 1.22]; protein: 0.41 a.u. [0.23 to 0.75] vs 0.29 a.u. [0.15 to 0.47]; data not shown). In addition, expression of MMP-9 mRNA (CONTROL vs SEVO: 0.72 a.u. [0.68 to 0.82] vs 0.70 a.u. [0.62 to 0.71]), MMP-2 mRNA (0.91 a.u. [0.91 to 0.99] vs 0.93 a.u. [0.89 to 0.97]; data not shown) and activity of MMP-9 (44.3 a.u. [33.3 to 45.7] vs 34.6 a.u. [23.6 to 62.4]) did not differ significantly, whereas MMP-2 activity was significantly decreased in the SEVO group (CONTROL vs SEVO: 34.8 a.u. [28.1 to 37.8] vs 22.0 a.u. [14.4 to 23.9]; *P *< 0.05).

## Discussion

Circulatory failure and myocardial dysfunction resulting from CA largely contribute to morbidity and mortality after initially successful CPR [19]. We have shown that pharmacological postconditioning with SEVO, when administered during reperfusion after successful CPR, improved myocardial dysfunction and reduced myocardial damage that was associated with attenuation of myocardial inflammation, apoptosis, increased HIF-1α expression and modulation of matrix remodeling. Based on the present protocol, early administration of SEVO did not, however, affect neurological recovery compared to propofol.

### Effects of sevoflurane on myocardial dysfunction and damage

Most experimental studies have documented improved cardiac performance when protective agents were given before the insult [20]. In patients with CA, however, pretreatment is virtually impossible because of the unpredictable onset. Therefore, as in our study, protective interventions should be started at the earliest time after the initiation of global reperfusion, when significant damage has already occurred. Zhao and coworkers [21], in an animal model of myocardial ischemia, demonstrated that ischemic postconditioning during reperfusion resulted in massive salvage of the myocardium and reduction of myocardial infarct size by 45%. In this context, pharmacological postconditioning with volatile anesthetics may offer an attractive opportunity to reduce organ damage in the postresuscitation period. In our study, SEVO administered instead of propofol during reperfusion after successful CPR attenuated serum troponin T release. Thus SEVO administered after ROSC reduced myocardial damage compared to propofol in the early postresuscitation period.

Moreover, postresuscitation myocardial dysfunction is one of the leading causes of early death after successful CPR [22]. Echocardiography-derived variables such as left ventricular ejection fraction and E/A ratio are used routinely for assessment of myocardial function. In addition, the echocardiographic myocardial performance index allows more sensitive and quantitative assessment of postresuscitation myocardial dysfunction [23]. In the present study, we found a deterioration of left ventricular performance in the initial postresuscitation period, but not 24 hours after ROSC. More interestingly, both left ventricular systolic and diastolic function were significantly impaired after successful CPR, which remained impaired in the CONTROL group but improved in the SEVO group during the initial postresuscitation period. After 24 hours of ROSC, however, no differences in systolic or diastolic function were detected. Upcoming studies must prove whether initial cardioprotective effects afforded by volatile anesthetics result in long-term beneficial effects. Russ *et al*. [24] also previously demonstrated, in a rat model of CA, that SEVO administered at the beginning of CPR was able to improve left ventricular ejection fraction, maximum cardiac power and end-diastolic volume within the first 3 hours after CPR. Since myocardial stunning has also previously been summarized as one potential underlying mechanism of postresuscitation myocardial dysfunction [22], attenuation of myocardial stunning may further be considered in terms of cardioprotective properties of SEVO postconditioning. In contrast, propofol is a widely used intravenous anesthetic agent with antioxidant properties secondary to its phenol-based chemical structure. From a clinical point of view, potential hemodynamic side effects (for example, hypotension) are reasonably comparable between propofol and SEVO. Mean arterial pressure did not differ between both groups in our study. It has been suggested that propofol itself may even provide dose-dependent cardiac protection, primarily by enhancing tissue antioxidant capacity and reducing lipid peroxidation [25,26]. Therefore, it is unlikely that the positive outcome after SEVO postconditioning might potentially be attributed to increased myocardial damage after propofol anesthesia.

Electrical instability with reperfusion ventricular arrhythmia commonly occurs after CPR and may further compromise postresuscitation survival. In the SEVO group in our study, the incidence of postresuscitation ventricular ectopic beats during the initial 30 minutes following successful CPR tended to be decreased compared to the CONTROL group, although statistical significance was not reached. The mechanism by which postconditioning with SEVO may reduce reperfusion-induced ventricular arrhythmias may be independent of known pathways that have been implicated in the infarct-sparing effects of pre- and postconditioning, including activation of adenosine, mitochondrial K_ATP _channel and mitochondrial permeability transition pore pathways, respectively.

### Effects of sevoflurane on myocardial apoptosis, inflammation and remodeling

Adrie *et al*. [3] hypothesized that postresuscitation disease may be related to an early systemic inflammatory response, leading to an exacerbation of the inflammatory balance. Stress-induced proinflammatory cytokines, particularly TNF-α and IL-1β synthesized and released in response to the stress of global ischemia accompanying CA, play a pivotal role in development of postresuscitation ventricular dysfunction [27]. In this respect, we found that expression of IL-1β tended to be reduced on the mRNA level and was statistically significantly reduced on the protein level in the SEVO group compared to the CONTROL group. In terms of inflammation, volatile anesthetics have been shown to reduce neutrophil adhesion in the reperfused coronary system and thereby preserve cardiac function [28]. Very recently, Mu *et al*. [29] reported that isoflurane protects against zymosan-induced generalized inflammation and associated lung injury in mice by enhancing the activities of antioxidant enzymes. Therefore, volatile anesthetics may provide a new adjuvant strategy for the treatment of critically ill patients. In parallel, pharmacological postconditioning might therefore also offer an attractive opportunity to ameliorate damage to vital organs in the postresuscitation period. Our data indicate that SEVO resulted in less Fas ligand expression that plays an important role in the regulation of apoptosis. In this respect, we found an increase in procaspase-3 in the SEVO group that could indicate less cleavage of this protein, suggesting less apoptosis in the SEVO group compared to CONTROL group. This is emphasized by several studies demonstrating that SEVO pre- and postconditioning decrease ischemia-induced apoptosis [30] by reduced activation of caspase-3 and caspase-9 mediated by phosphorylation of Akt and extracellular signal-related kinases [31], as well as less release of cytochrome *c *and caspase-3 activation mediated by increased Bcl-2 expression and activation of NF-κB [32].

Eckle *et al*. [33] provided evidence for a critical role of HIF-1α in cardioprotection by ischemic preconditioning. In addition, Zhao and colleagues [34] suggested recently that HIF-1α is also involved in ischemic postconditioning, and pharmacological augmentation of HIF-1α expression may even enhance the myocardial infarct-sparing effect. In the present study, expression of HIF-1α was increased on mRNA and protein levels in the SEVO group compared to the CONTROL group. Thus our data confirm the results of several studies that found upregulation of HIF-1α after preconditioning with volatile anesthetics in both *in vitro *[35,36] and *in vivo *[37] models of ischemia-reperfusion injury.

After substantial myocardial ischemia, major tissue remodeling occurs to restore the structural architecture and cardiac function. These remodeling events are parallel to increased expression and activity of MMPs in the postinfarct myocardium [38]. The interaction between global ischemia following CA, matrix remodeling and volatile anesthetics has been poorly studied to date.

Regarding the effect of SEVO on myocardial tissue remodeling, we found that SEVO increased MMP-9 activity in myocardial tissue, whereas the effects on MMP-2 activity were indistinguishable. Since IL-1β and other proinflammatory cytokines may also be actively involved in the regulation of matrix remodeling processes after myocardial ischemia [39], the issue of tissue remodeling and the regulatory effect of IL-1β on MMP activity could be very important. Interestingly, *in vitro *data from our group points toward positive regulatory effects of IL-1β on MMP-2 and MMP-9 expression and activity [40]. These data suggest that the SEVO-induced attenuation of IL-1β expression in the present study might affect myocardial MMP-2 and MMP-9 activity in such a way as to facilitate tissue remodeling following global ischemia and CPR.

### Effects of sevoflurane on neurological function, cerebral apoptosis and inflammation

Volatile anesthetics also possess a variety of neuroprotective mechanisms of action that include inhibition of spontaneous depolarization in the ischemic penumbra, antioxidant potential and *N*-methyl-D-aspartate receptor antagonism after cerebral ischemia [41,42]. For example, Blanck *et al*. [43] demonstrated functional reduction in neurological injury following 8 minutes of CA after SEVO-induced preconditioning. In the same way, isoflurane proved to be effective in rodent models of middle cerebral artery occlusion, causing increased tolerance of the brain to a subsequent ischemic insult [41]. In contrast to our study, these observations were all based on pretreatment, which limits SEVO's clinical applicability, at least for stroke and CA patients, in whom symptoms appear suddenly and unexpectedly. Our results, however, suggest that SEVO administered during reperfusion after successful CPR does not promote beneficial effects, at least in terms of neurological deficits, cerebral inflammation and apoptosis in this experimental model. The absence of any neuroprotective effects of SEVO may theoretically be rooted in the condition that CA time was to short in our study, resulting in moderate cerebral ischemia and low deficit scores. Indeed, Fries *et al*. [44] previously reported higher NDSs and cerebral alterations in a porcine model of 8-minute CA, but very comparably to our data, administration of the volatile anesthetic isoflurane after CPR did not improve neurological deficits, neurocognitive function and ischemic damage of neurons in the CA1 sector of the hippocampus. Since ischemic damage in brain tissue may occur much earlier than in myocardial tissue, studying only one protocol with one type of severity of ischemic damage to the brain cannot exclude whether, with longer or shorter periods of global ischemia, SEVO might provide neuroprotection. The present study results with 8 minutes of CA, however, show that early administration of volatile anesthetics after CPR may not improve neurological functional outcome and therefore may be of limited value for the preservation of neurological function.

### Limitations

There are some points that need to be addressed. First, mild therapeutic hypothermia has emerged as an effective strategy to reduce neurological impairment after successful CPR [45]. To account for temperature dependencies, however, normothermic body temperature was maintained in both the CONTROL and SEVO groups in the present study. Potential protective effects of volatile anesthetics depend on energy-dependent signal transduction, for example, protein synthesis and phosphorylation that may be otherwise affected by hypothermia-induced decrease of global metabolic rate as well as suppression of protein synthesis. Second, our present study is powered for a cardiovascular end point to detect a 25% difference in left ventricular ejection fraction. Therefore, the analysis of neurological outcome differences may be limited with this group size, although we previously demonstrated neuroprotective effects by induction of mild therapeutic hypothermia with a comparable global ischemia time [46]. Third, exposure to SEVO longer than 4 hours might have had beneficial effects in neurological recovery. Fourth, a few studies have demonstrated that, to be successful, postconditioning should be initiated in the very initial minutes of reperfusion. Therefore, we cannot exclude that SEVO had any postconditioning effect in our study, because SEVO was started 10 minutes after stable, unassisted circulation. Blinding the investigator was not possible throughout the experiment because of different techniques of drug administration (intravenous versus volatile anesthetics), but echocardiographic variables and serum and tissue samples were analyzed in a blinded fashion. Finally, postresuscitation care was standardized up to 4 hours after ROSC, but not thereafter, and may have affected the results 24 hours after ROSC.

## Conclusions

Pharmacological postconditioning with the volatile anesthetic SEVO, when administered during reperfusion after successful CPR, reduced myocardial damage and improved myocardial dysfunction associated with attenuation of myocardial inflammation, apoptosis, increased HIF-1α and modulation of matrix remodeling. On the basis of the protocol we used, we found that early administration of SEVO may not improve neurological recovery over propofol administration.

## Key messages

• Postresuscitation myocardial dysfunction is a critical issue that contributes to the so-called postresuscitation disease in patients with successful CPR.

• Pharmacological postconditioning with the volatile SEVO, when administered during early reperfusion after successful CPR, reduces myocardial damage and myocardial dysfunction in the early period after CPR.

• In addition, SEVO is associated with reduced myocardial proinflammatory cytokine expression, apoptosis, increased HIF-1α expression and increased activity of MMP-9.

• Based on the present protocol, early administration of SEVO, however, may not improve neurological recovery compared to propofol anesthesia

## Abbreviations

CA: cardiac arrest; CONTROL: control group; CPR: cardiopulmonary resuscitation; ECG: electrocardiogram; ELISA: enzyme-linked immunosorbent assay; HIF: hypoxia-inducible factor; IL: interleukin; MMP: matrix metalloproteinase; NDS: neurological deficit score; NF-κB: nuclear factor κB; ROSC: return of spontaneous circulation; SEVO: sevoflurane; TNF-α: tumor necrosis factor α; VF: ventricular fibrillation.

## Competing interests

The authors declare that they have no competing interests. This paper is not currently under consideration for publication elsewhere and has not been published previously in any form. This work was accepted in part for poster presentation at the American Heart Association annual meeting, November 2010, Chicago, IL, USA.

## Authors' contributions

PM, MG, MA, JS and BB conceived the study and designed the trial. PM, MG and BB obtained research funding. MA, KZ, MM, NF, CM and ST performed experimental and laboratory analysis. JS provided statistical advice on study design. PM drafted the manuscript, and all authors contributed substantially to its revision. PM and BB takes responsibility for the paper as a whole.

## Supplementary Material

Additional file 1 **Supplemental digital content: methods S1**. Microsoft Word file containing detailed information about echocardiography, Western blot analysis, semiquantitative RT-PCR and gelatin zymography. Click here for file

Additional file 2 **Supplemental digital content: methods S2**. Microsoft Word file containing two Tables S1 and S2, which provide detailed information about swine used in the study with neurological deficit scores (NDSs) 1 and 2. Click here for file
