# Supplementary material for: Pharmacological postconditioning with sevoflurane after cardiopulmonary resuscitation reduces myocardial dysfunction
Source: Crit Care. 2011 Oct 19;15(5):R241. doi: 10.1186/cc10496 (PMC3334792; doi:10.1186/cc10496)
Supplement: Additional file 1 — Supplemental digital content: methods S1. Microsoft Word file containing detailed information about echocardiography, Western blot analysis, semiquantitative RT-PCR and gelatin zymography. [file cc10496-S1.DOC]

# Pharmacological postconditioning with sevoflurane after cardiopulmonary resuscitation reduces myocardial dysfunction

Supplemental Digital Content - Methods S1

**Echocardiography**

Mitral valve inflow velocity pattern was recorded from the midesophageal four-chamber view with the pulsed-wave Doppler sample volume positioned at the tips of the mitral leaflets during diastole. Peak velocities of transmitral inflow were measured in early (E) and late (A) diastole, and the E/A ratio was calculated. Left ventricle outflow tract and the ascending aorta were imaged, and a pulsed wave Doppler was positioned at the LV outflow tract with the sample volume just below the aortic valve for determination of ejection time. Beam position and gain settings were optimized to achieve the greatest amplitude and clarity of the Doppler spectrum. Left ventricle ejection time was measured from the beginning to the end of the aortic flow wave. The myocardial performance index has been developed to reflect systolic and diastolic function [1]. The interval (a) was measured from mitral valve closing to opening, which is equal to the sum of isovolumetric contraction time, ejection time, and isovolumetric relaxation time. Left ventricle ejection time (b) was measured from the onset to the end of blood flow in the left ventricle outflow tract. The sum of isovolumetric contraction time and isovolumetric relaxation time was obtained by subtracting (b) from (a). Myocardial performance index was then calculated as ((a- b)/ b). All echocardiographic measurements were recorded as the mean of three consecutive cardiac cycles, and were registered according to the recommendations of the American Society of Echocardiography [2]. Another experienced echocardiographer blinded to group assignment performed *post hoc* off-line quantitative data analyses.

**Western blotting**

Two different protocols employing RIPA buffer and SDS, respectively, were used for the preparation of tissue samples. (i) protein extraction using RIPA buffer: Samples of frozen myocardial or cerebral tissue (30 mg) were mixed with 800 µl RIPA buffer including protease and phosphatase inhibitors (Roche, Mannheim, Germany) and homogenized on ice with 10-15 strokes using a teflon-glass homogenizer. The solution was centrifuged for 2 minutes at 4°C and 300x*g* using QIAshredder filters (Qiagen) and was stored at -20°C until use. (ii) protein extraction using SDS: Tissue samples were homogenized as described above in a buffer containing 2% sodium dodecyl sulfate (SDS), 10 mM Tris-HCl (pH 7.4) freshly supplemented with sodium fluoride (10 mM), sodium pyrophosphate (10 mM), sodium orthovanadate (1mM), sodium molybdate (1 mM), phenylarsine oxide (1 M), and aprotinin (10 μg/ml). The homogenate obtained by both methods was boiled for 5 minutes after addition of SDS-polyacrylamide gel electrophoresis (PAGE) sample buffer. Protein concentrations were determined with a BCA Protein Assay kit (Pierce Biotechnology, Rockford, USA). An equal amount of protein (30 μg) of each sample was separated by 12% SDS-PAGE and transferred onto a nitrocellulose membrane (Amersham Pharmacia Biotech, Piscataway, USA). The membranes were then incubated in a blocking solution (Starting Block T20; Thermo scientific, Rockford, USA) containing 5% non-fat milk powder for 1 hour at room temperature followed by overnight incubation with specific antibodies for procaspase-3 (Cell signaling Technology, Danvers, USA; dilution 1:200), hypoxia inducible factor (HIF)-1α (Novus Biologicals, Littleton, USA; 1:3,500) and β-actin (Santa Cruz, Heidelberg, Germany; dilution 1:1,000), which served as a loading control. After washing in PBS containing 0.05% Tween 20 (PBS-T; Sigma-Aldrich, St. Louis, USA), membranes were incubated for 1 hour with peroxidase-conjugated goat anti-rabbit immunoglobulin G (DAKO, Glostrup, Denmark, dilution 1:10,000) or with peroxidase-conjugated rabbit anti-goat immunoglobulin G (Santa Cruz, Heidelberg, Germany; dilution 1:1,000). The final reaction was visualized using enhanced chemiluminescence (ECL Western Blotting Detection Reagents, Amersham Pharmacia Biotech, Piscataway, USA), and the membranes were exposed to x-ray film. Bands were scanned and the optical density was quantified with densitometry and expressed as the ratio of procaspase-3 to β-actin and HIF-1α to β-actin, respectively.

**Semiquantitative RT-PCR**

Isolation of RNA was performed with the Qiagen RNeasy minikit according to the manufacturer’s protocol. RNA concentrations were quantified with a spectrophotometer at 260nm, purity of RNA was assessed by the 260/280nm ratio. 200ng of total RNA were used to produce cDNA by a reverse transcription Kit (Applied Biosystems, California, USA) employing random hexamer primers and 2µl of the resulting cDNA were employed as template for further PCR experiments in a final volume of 20µl. All PCR experiments were performed with DNA Taq Polymerase from Solis BioDyne, Tartu, Estonia. The following primers were synthesized (Metabion, Martinsried, Germany) and employed to amplify specific fragments of the pig transcripts: interleukin (IL)-1β (NM_214055.1) 5´-TGTCTGTCATCGTGGCAGTGGA-3´ and 5´-TTGTTGCTATCATCTCCTTGCAC -3´, annealing temperature 58°C, amplicon size 307bp; Fas-ligand (NM_213806.1) 5´-AAGAGGGACCACAATGCAGG-3´ and 5´-AGCTCCTTTTTCTCAGAGGGTAGA-3´, annealing temperature 58°C, amplicon size 209bp; caspase-3 (NM_214131) 5´-GCTGCAAATCTCAGGGAGAC-3´ and 5´-GGCAGGCCTGAATTATGAAA-3´, annealing temperature 58°C, amplicon size 280bp; HIF-1α (NM_001123124) 5´-TTTAACTTTGTTGGCCCCAG-3´ and 5´-CCAATGGTGACAACTGATCG-3´, annealing temperature 58°C, amplicon size 566bp; MMP-2 (AF295805) 5´-GACAGTGACACCACGTGAC-3´ and 5´-CAGGCGTCTGCAATGAGCT-3´, annealing temperature 58°C, amplicon size 345bp; MMP-9 ([NM_001038004](http://www.ncbi.nlm.nih.gov/entrez/viewer.fcgi?db=nuccore&id=83921636)) 5´-GGACGCCAAGTGTGGGTG-3´ and 5´-GTCCACCTGATTCACCTCGT-3´, annealing temperature 58°C, amplicon size 326bp; GAPDH (AF017079) 5´-ATTGCCCTCAACGACCACT-3´ and 5´-GGCCTCTCTCCTCCTCGC-3´, annealing temperature 58°C, amplicon size 159bp. Negative controls were performed by omitting the respective input cDNA. PCR products were separated on 2.5% of agarose gels, followed by ethidiumbromide staining and were visualized by UV-transilumination. Images were taken and densitometrically analysed with the software ImageJ (v1.41o, NIH).

**Gelatine zymography of MMP-9 and MMP-2**

Zymography was performed as described previously [3]. For detection of matrix metalloproteinase (MMP) activity, myocardial and cerebral tissue samples were homogenized in RIPA buffer (for details see Western blotting). Following a 1 minute centrifugation step at 1,000x*g*, supernatants were stored at –20°C until use. 30 µg protein was loaded and separated on 7 % SDS – page gels (containing 1 mg/ml gelatine) under non-reducing conditions. After electrophoresis the gels were soaked in 2.5 % Triton X-100 for 30 minutes to remove SDS and incubated in Tris-HCl (50 mmol/l, pH 7.5), containing CaCl_2_ (5 mmol/l), and ZnCl_2_ (1 mmol/l) overnight at 37°C. After Coomassie blue staining white bands of lysis indicated digestion of gelatine by MMPs. Densitometric analysis was performed using the ImageJ 1.41 software (ImageJ, NIH, USA).

**References**

1. Tei C, Ling LH, Hodge DO, Bailey KR, Oh JK, Rodeheffer RJ, Tajik AJ, Seward JB: **New index of combined systolic and diastolic myocardial performance: a simple and reproducible measure of cardiac function--a study in normals and dilated cardiomyopathy**. *J Cardiol* 1995, **26**(6):357-366.

2. Gottdiener JS, Bednarz J, Devereux R, Gardin J, Klein A, Manning WJ, Morehead A, Kitzman D, Oh J, Quinones M *et al*: **American Society of Echocardiography recommendations for use of echocardiography in clinical trials**. *J Am Soc Echocardiogr* 2004, **17**(10):1086-1119.

3. Kleiner DE, Stetler-Stevenson WG: **Quantitative zymography: detection of picogram quantities of gelatinases**. *Anal Biochem* 1994, **218**(2):325-329.
