# Supplementary material for: Pharmacological postconditioning with sevoflurane after cardiopulmonary resuscitation reduces myocardial dysfunction
Source: Crit Care. 2011 Oct 19;15(5):R241. doi: 10.1186/cc10496 (PMC3334792; doi:10.1186/cc10496)
Supplement: Additional file 2 — Supplemental digital content: methods S2. Microsoft Word file containing two Tables S1 and S2, which provide detailed information about swine used in the study with neurological deficit scores (NDSs) 1 and 2. [file cc10496-S2.DOC]

# Pharmacological postconditioning with sevoflurane after cardiopulmonary resuscitation reduces myocardial dysfunction

Supplemental Digital Content - Methods S2

**Table S1. Swine neurologic deficit score I**

| **Consciousness** | 0 = Normal | 10 = Clouded | 25 = Stupor/Coma |
| --- | --- | --- | --- |
| **Respiration** | 0 = Normal | 10 = Abnormal | 25 = Apnea |
| **Posture** | 0 = Normal | 10 = Atactic | 25 = Cannot stand or walk |
| **Food and water intake** | 0 = Normal | 10 = Irregular | 25 = Does not eat or drink |

Neurologic deficit score I according to Tang and colleagues [1].

**Table S2. Swine n**eurologic deficit score II

| **Level of consciousness** | 0 = Normal: Complete awareness of auditory stimuli | 30 = Clouded: conscious, but drowsy or irritable | 60 = Stupor: motor response only to painful stimuli | 100 = Coma: no motor response to painful stimuli |
| --- | --- | --- | --- | --- |
| **Respiratory pattern** | 0 = Normal | 50 = Abnormal spontaneous breathing | 100 = Apnea |  |
| **Motor and sensory function** |  |  |  |  |
| Motor response to painful stimulus: pinch hoof-pad | 0 = Normal: brisk withdrawal | 10 = Sluggish response | 25 = Very sluggish response | 50 = No response |
| Muscle tone: pick up and release extremity | 0 = Normal tone | 25 = 1 or 2 extremities stiff or flaccid | 50 = 3 or 4 extremities stiff or flaccid |  |
| **Behavior** |  |  |  |  |
| Standing | 0 = Can stand | 20 = Cannot stand |  |  |
| Walking | 0 = Normal | 10 = Unsteady gait | 20 = Very unsteady or ataxic gait | 30 = Cannot walk |
| Restraint: attempt to hold down pig from behind | 0 = Normal: vigorously resists | 20 = Sluggish: resists | 40 = Very sluggish: resists minimally | 50 = No resistance |

Neurologic deficit score II according to Berg and colleagues [2].

**References**

1. Tang W, Weil MH, Schock RB, Sato Y, Lucas J, Sun S, Bisera J: **Phased chest and abdominal compression-decompression. A new option for cardiopulmonary resuscitation**. *Circulation* 1997, **95**(5):1335-1340.

2. Berg RA, Otto CW, Kern KB, Sanders AB, Hilwig RW, Hansen KK, Ewy GA: **High-dose epinephrine results in greater early mortality after resuscitation from prolonged cardiac arrest in pigs: a prospective, randomized study**. *Crit Care Med* 1994, **22**(2):282-290.
